# A Deep Graph Network–Enhanced Sampling Approach to Efficiently Explore the Space of Reduced Representations of Proteins

**DOI:** 10.3389/fmolb.2021.637396

**Published:** 2021-04-29

**Authors:** Federico Errica, Marco Giulini, Davide Bacciu, Roberto Menichetti, Alessio Micheli, Raffaello Potestio

**Affiliations:** ^1^ Department of Computer Science, University of Pisa, Pisa, Italy; ^2^ Physics Department, University of Trento, Trento, Italy; ^3^ INFN-TIFPA, Trento Institute for Fundamental Physics and Applications, Trento, Italy

**Keywords:** molecular dynamics, coarse-grained methods, mapping entropy, deep learning, neural networks for graphs, neural networks

## Abstract

The limits of molecular dynamics (MD) simulations of macromolecules are steadily pushed forward by the relentless development of computer architectures and algorithms. The consequent explosion in the number and extent of MD trajectories induces the need for automated methods to rationalize the raw data and make quantitative sense of them. Recently, an algorithmic approach was introduced by some of us to identify the subset of a protein’s atoms, or mapping, that enables the most informative description of the system. This method relies on the computation, for a given reduced representation, of the associated mapping entropy, that is, a measure of the information loss due to such simplification; albeit relatively straightforward, this calculation can be time-consuming. Here, we describe the implementation of a deep learning approach aimed at accelerating the calculation of the mapping entropy. We rely on Deep Graph Networks, which provide extreme flexibility in handling structured input data and whose predictions prove to be accurate and-remarkably efficient. The trained network produces a speedup factor as large as 10^5^ with respect to the algorithmic computation of the mapping entropy, enabling the reconstruction of its landscape by means of the Wang–Landau sampling scheme. Applications of this method reach much further than this, as the proposed pipeline is easily transferable to the computation of arbitrary properties of a molecular structure.

## Introduction

Molecular dynamics (MD) simulations (Alder and Wainwright, 1959; Karplus, 2002) are an essential and extremely powerful tool in the computer-aided investigation of matter. The usage of classical, all-atom simulations has boosted our understanding of a boundless variety of different physical systems, ranging from materials (metals, alloys, fluids, etc.) to biological macromolecules such as proteins. As of today, the latest software and hardware developments have pushed the size of systems that MD simulations can address to the millions of atoms (Singharoy et al., 2019), and the time scales covered by a single run can approach the millisecond for relatively small molecules (Shaw et al., 2009).

**FIGURE 1:**
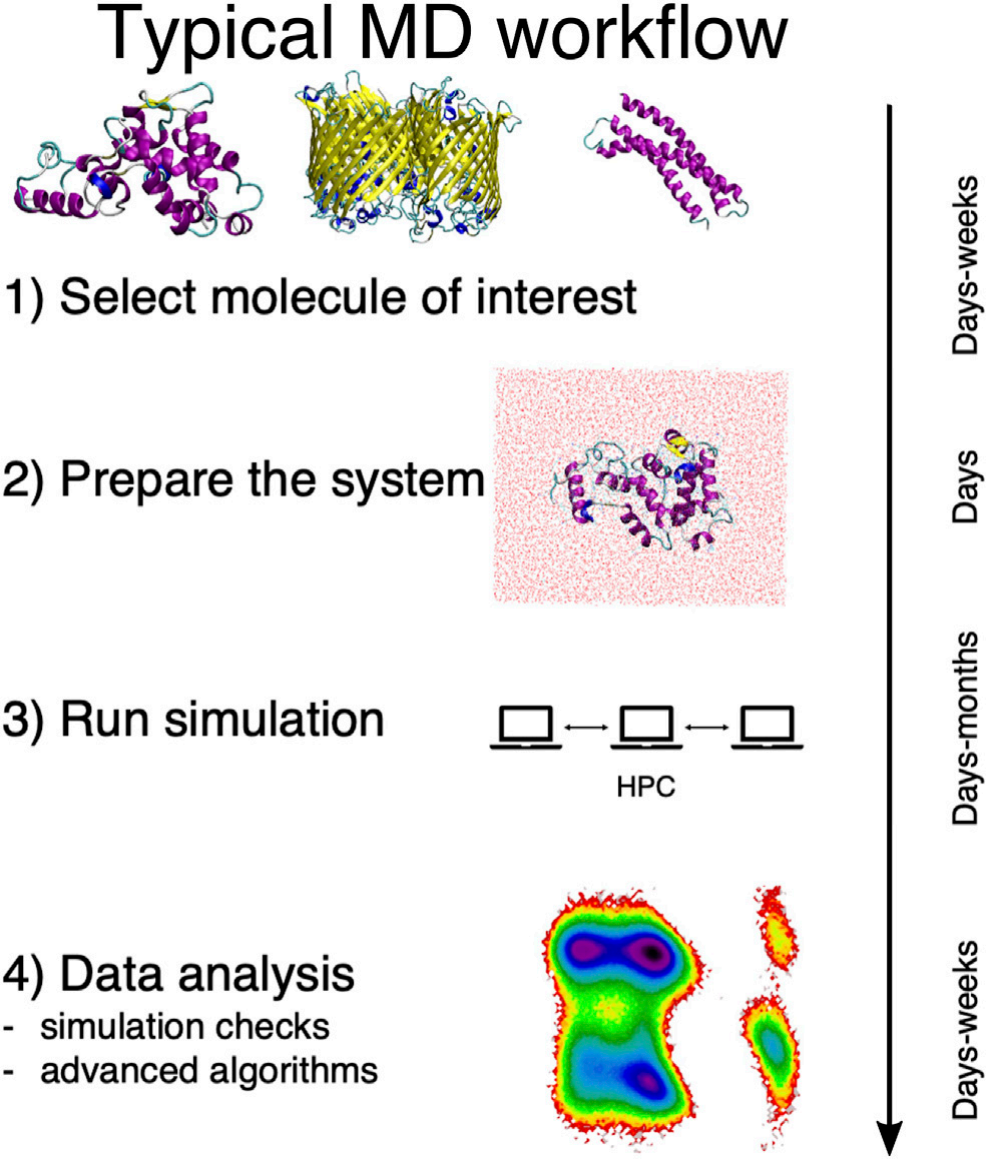
Schematic representation of the typical workflow of a molecular dynamics study. On the right we report the average time scales required for each step of the process.

In general, a traditional MD-based study proceeds in four steps, here schematically summarized in Figure 1. First, the system of interest has to be identified; this apparently obvious problem can actually require a substantial effort *per se*, e.g., in the case of dataset-wide investigations. Second, the simulation setup has to be constructed, which is another rather nontrivial step (Kandt et al., 2007). Then the simulation has to be run, typically on a high performance computing infrastructure. Finally, the output has to be analyzed and rationalized *in order to extract information from the data*.

This last step is particularly delicate, and it is acquiring an ever growing prominence as large and long MD simulations can be more and more effortlessly performed. The necessity thus emerges to devise a parameter-free, automated “filtering” procedure to describe the examined system in simpler, intelligible terms and make sense of the immense amount of data we can produce—but not necessarily understand.

In the field of soft and biological matter, coarse-graining (CG) methods represent a notable example of a systematic procedure that aims at extracting, out of a detailed model of a given macromolecular system, the relevant properties of the latter (Marrink et al., 2007; Takada, 2012; Saunders and Voth, 2013; Potestio et al., 2014). This is achieved through the construction of simplified representations of the system that have fewer degrees of freedom with respect to the reference model while retaining key features and properties of interest. In biophysical applications, this amounts to describing a biomolecule, such as a protein, using a number of constituent units, called CG sites, lower than the number of particles composing the original, atomistic system.

**FIGURE 2:**
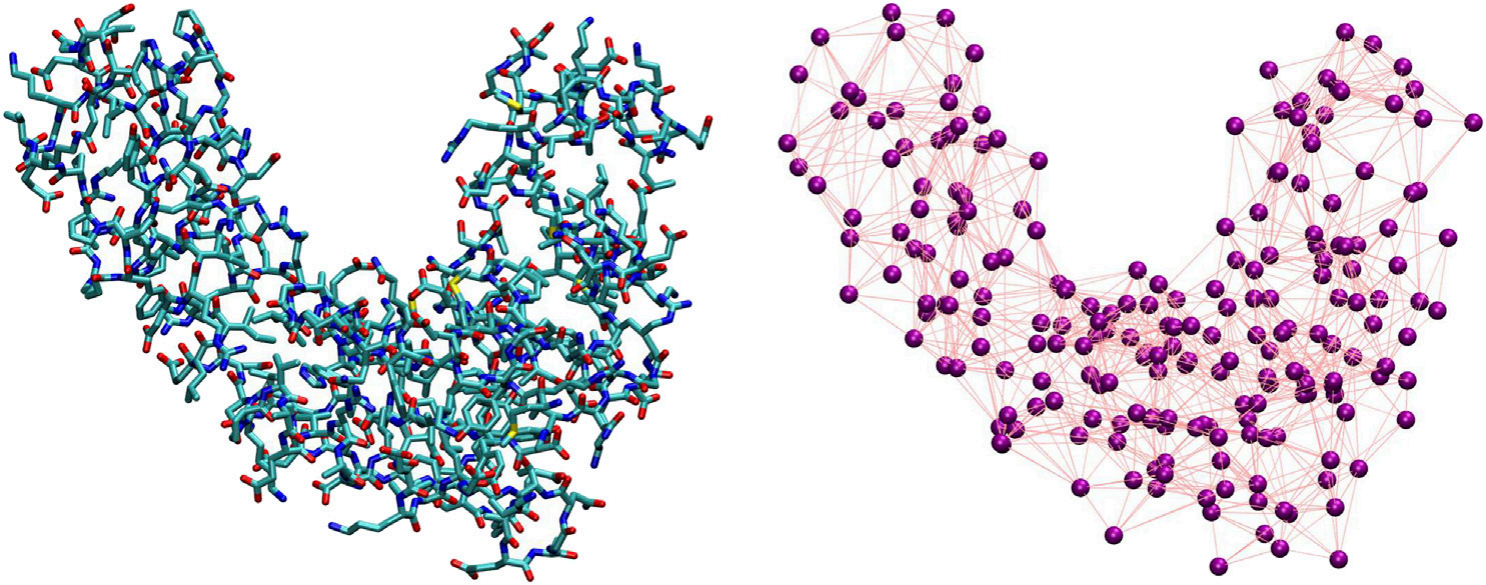
Comparison between an all-atom, detailed description of a protein **(left)** and one of its possible coarse-grained representations **(right)**. The purple spheres on the right plot correspond to CG sites, while the edges connecting them represent the effective interactions.

The coarse-graining process in soft matter requires two main ingredients, separately addressing two entangled, however conceptually very different, problems (Noid, 2013a). The first ingredient consists of the definition of a *mapping*, that is, the transformation **M**(**r**) = **R** that connects a high-resolution representation **r** of the system’s configuration to a low-resolution one **R**. The mapping thus pertains to the *description* of the system’s behavior,“filtered” so as to retain only a subset of the original degrees of freedom. The second ingredient is the set of effective interactions introduced among the CG sites; these CG potentials serve the purpose of *reproducing a posteriori* the emergent properties of the system directly from its simplified representation rather than from its higher-resolution model. Both ingredients are highlighted in Figure 2, where we display a visual comparison between a high-resolution representation of a protein and one among its possible simplified depictions, as defined by a particular selection of the molecule’s retained atoms.

During the past few decades, substantial effort has been invested in the correct parameterization of CG potentials (Noid et al., 2008; Shell, 2008; Noid, 2013b): most of the research focused on accurately reproducing the system’s behavior that arises from a model relying on a specific choice of the CG observational filter. Critically, the investigation of the quality of the filter *itself*—that is, the definition of the CG mapping—has received much less attention. Indeed, most methods developed in the field of soft matter do not make use of a system-specific, algorithmic procedure for the selection of the effective sites but rather rely on general criteria, based on physical and chemical intuition, to group together atoms in CG “beads” irrespective of their local environment and global thermodynamics (Kmiecik et al., 2016)—one notable example being the representation of a protein in terms of its α-carbon atoms.

While acceptable in most practical applications, this approach entails substantial limitations: in fact, the CG process implies a loss of information and, through the application of universal mapping strategies, system-specific properties, albeit relevant, might be “lost in translation” from a higher to a lower resolution representation (Foley et al., 2015; Jin et al., 2019; Foley et al., 2020). Hence, a method would be required that enables the automated identification of which subset of retained degrees of freedom of a given system preserves the majority of important detail from the reference, while at the same time reducing the complexity of the problem. In the literature, this task has been addressed through several different techniques, such as graph-theoretical analyses (Webb et al., 2019), geometric criteria (Bereau and Kremer, 2015), and machine learning algorithms (Murtola et al., 2007; Wang and Bombarelli, 2019; Li et al., 2020). These efforts are rooted in the assumption that the optimal CG representation of a system can be determined solely by exploiting a subset of features of the latter. In contrast, taking into account the full information content encoded in the system requires statistical mechanics-based models, where the optimal CG mapping is expected to emerge systematically from the comparison between the CG model and its atomistic counterpart. Within this framework, pioneering works rely on a simplified description of the system (Koehl et al., 2017; Diggins et al., 2018), e.g., provided by analytically solvable, linearized elastic network models, which cannot faithfully reproduce the complexity of the *true* interaction network.

A recently developed statistical mechanics-based strategy that aims at overcoming such limitations is the one relying on the minimization of the mapping entropy (Giulini et al., 2020), which performs, in an unsupervised manner, the identification of the subset of a molecule’s atoms that retains the largest possible amount of information about its behavior. This scheme relies on the calculation of the mapping entropy *S*_*map*_ (Shell, 2008; Rudzinski and Noid, 2011; Shell, 2012; Foley et al., 2015), a quantity that provides a measure of the dissimilarity between the probability density of the system configurations in the original, high-resolution description and the one marginalized over the discarded atoms. *S*_*map*_ is employed as a cost function and minimized over the possible reduced representations so as to systematically single out the most informative ones.

The method just outlined suffers from two main bottlenecks: on the one hand, the determination of the mapping entropy is *per se* computationally intensive; even though smart workarounds can be conceived and implemented to speed up the calculation, its relative complexity introduces a nontrivial slowdown in the minimization process. On the other hand, the sheer size of the space of possible CG mappings of a biomolecule is so ridiculously large that it makes a random search practically useless and an exhaustive enumeration simply impossible. Hence, an optimization procedure is required to identify the simplified descriptions that entail the largest amount of information about the system. Unfortunately, this procedure nonetheless implies the calculation of *S*_*map*_ over a very large number of tentative mappings, making the optimization, albeit possible, computationally intensive and time consuming.

In this work, we present a novel computational protocol that suppresses the computing time of the optimization procedure by several orders of magnitude, while at the same time boosting the sampling accuracy. This strategy relies on the fruitful, and to the best of our knowledge unprecedented combination of two very different techniques: graph-based machine learning models (Micheli, et al., 2009; Bronstein et al., 2017; Hamilton et al., 2017; Battaglia et al., 2018; Zhang et al., 2018; Zhang et al., 2019; Bacciu et al., 2020; Wu et al., 2021) and the Wang–Landau enhanced sampling algorithm (Wang and Landau, 2001a; Wang and Landau, 2001b; Shell et al., 2002; Barash et al., 2017). The first serves the purpose of reducing the computational cost associated with the estimation of the mapping entropy; the second enables the efficient and thorough exploration of the mapping space of a biomolecule.

An essential element of the proposed method is thus a graph-based representation of our object of interest, namely a protein. With their long and successful story both in the field of coarse-graining (Gfeller and Rios, 2007; Webb et al., 2019; Li et al., 2020) and in the prediction of protein properties (Borgwardt et al., 2005; Ralaivola et al., 2005; Micheli et al., 2007; Fout et al., 2017; Gilmer et al., 2017; Torng and Altman, 2019), graph-based learning models represent a rather natural and common choice to encode the (static) features of a molecular structure; here, we show that a graph-based machine learning approach can reproduce the results of mapping entropy estimate obtained by means of a much more time-consuming algorithmic workflow. To this end, we rely on Deep Graph Networks (DGNs) (Bacciu et al., 2020), a family of machine learning models that learn from graph-structured data, where the graph has a variable size and topology; by training the model on a set of tuples (protein, CG mapping, and *S*_*map*_), we can infer the *S*_*map*_ values of unseen mappings associated with the same protein making use of a tiny fraction of the extensive amount of information employed in the original method, i.e., the molecular structure viewed as a graph. Compared to the algorithmic workflow presented in Giulini et al. (2020), the trained DGN proves capable of accurately calculating the mapping entropy arising from a particular selection of retained atoms throughout the molecule in a negligible time.

This computational speedup can be leveraged to perform a thorough, quasi-exhaustive characterization of the mapping entropy landscape in the space of possible CG representations of a system, a notable advancement with respect to the relatively limited exploration performed in Giulini et al. (2020). Specifically, by combining inference of the DGNs with the Wang–Landau sampling technique, we here provide an estimate of the density of states associated with the *S*_*map*_, that is, the number of CG representations in the biomolecule mapping space that generate a specific amount of information loss with respect to the all-atom reference. A comparison of the WL results on the DGNs with the exact ones obtained from a random sampling of mappings shows that the machine learning model is able to capture the correct population of CG representations in the *S*_*map*_ space. This analysis further highlights the accuracy of the model in predicting a complex observable such as the mapping entropy, which in principle depends on the whole configurational space of the macromolecule, only starting from the sole knowledge of the static structure of the latter.

## Materials and Methods

In this section, we outline the technical ingredients that lie at the basis of the results obtained in this study. Specifically, in *Mapping entropy* we summarize the mapping entropy protocol for optimizing CG representations presented in Giulini et al. (2020); in *Protein structures and data sets* we briefly describe the two proteins analyzed in this work as well as the data sets fed to the machine learning architecture; in *Data Representation and Machine Learning model* we illustrate our choice for the representation of the input data, together with theoretical and computational details about DGNs; finally, in *Wang–Landau Sampling* we describe our implementation of the Wang–Landau sampling algorithm as applied to the reconstruction of the mapping entropy landscape of a system.

### Mapping Entropy

The challenge of identifying maximally informative CG representations for a biomolecular system has been recently tackled by some of us (Giulini et al., 2020); specifically, we developed an algorithmic procedure to find the mappings that minimize the amount of information that is lost when the number of degrees of freedom with which one observes the system is *decimated*, that is, a subset of its atoms is retained while the remainder is integrated out. The quantity that measures this loss is called mapping entropy *S*_*map*_ (Shell, 2008; Rudzinski and Noid, 2011; Shell, 2012; Foley et al., 2015), which in the case of decimated CG representations can be expressed as a Kullback–Leibler divergence D_KL_ (Kullback and Leibler, 1951) between two probability distributions (Rudzinski and Noid, 2011),

$$S_{map}=k_{B}\times D_{KL}\left(p_{r}\left(r\right)||{\bar{p}}_{r}\left(r\right)\right)=k_{B}\int dr\;p_{r}\left(r\right)\text{ln}\left[\frac{p_{r}\left(r\right)}{{\bar{p}}_{r}\left(r\right)}\right].$$ (1)

Here, $p_{r}\left(r\right)$ is the probability of sampling a configuration **r** in the high-resolution description, namely, the Boltzmann distribution $p_{r}\left(r\right)\propto \text{exp}\left[-\beta u\left(r\right)\right]$, where u(r) is the atomistic potential and $\beta =1/k_{B}T$ is the inverse temperature. ${\bar{p}}_{r}\left(r\right)$, on the other hand, is the distribution obtained by observing the system through the “coarse-graining grid,” i.e., in terms of the selected CG mapping. ${\bar{p}}_{r}\left(r\right)$ is defined as (Rudzinski and Noid, 2011)

$${\bar{p}}_{r}\left(r\right)=p_{R}\left[M\left(r\right)\right]/{\text{Ω}}_{1}\left[M\left(r\right)\right],$$ (2)

where

$$p_{R}\left(R\right)=\frac{1}{Z}\int dre^{-\beta u\left(r\right)}\delta \left[M\left(r\right)-R\right]$$ (3)

is the probability of sampling the configuration R=M(r) in the low-resolution description—*Z* being the canonical partition function of the system—while

$${\text{Ω}}_{1}\left(R\right)=\int dr\;\delta \left(M\left(r\right)-R\right)$$ (4)

is the number of microstates **r** that map onto the CG configuration **R**.

The mapping entropy quantifies the information loss one experiences by replacing the original, microscopic distribution $p_{r}\left(r\right)$ of the system by an effective one in which the probability of a CG macrostate is equally redistributed to all microstates that map onto it. It follows that different choices of the CG mapping lead to different ${\bar{p}}_{r}\left(r\right)$ and, consequently, to different amounts of information losses arising from CG’ing.

The definition in Eq. 1 does not allow, given a CG representation, to directly determine the associated mapping entropy. It is however possible to perform a cumulant expansion of Eq. 1; by doing so, Giulini et al. (2020) showed that *S*_*map*_ can be approximately calculated as a weighted average over all CG macrostates **R** of the variances of the atomistic potential energies of all configurations **r** that map onto a specific macrostate. This strategy enables one to measure *S*_*map*_ only provided a set of all-atom configurations sampled from $p_{r}\left(r\right)$ and a decimation mapping.

The following, natural step in the analysis is then to identify the reduced representations of a system that are able to preserve the maximum amount of information from the all-atom reference—i.e., which minimize the mapping entropy. However, for a molecule with *n* atoms, the number of possible decimation mappings is 2^n^, an astronomical amount even for the smallest proteins. This number remains huge even narrowing down the exploration to a fixed number of retained atoms *N*, so that n!/[N! (n−N)!] mappings can be constructed. As a complete enumeration of all possible CG representations of a system is unfeasible in practice, Giulini et al. (2020) relied on a stochastic minimization procedure to extract a pool of optimized solutions out of this immense space, namely a simulated annealing approach (Kirkpatrick et al., 1983; Černỳ, 1985) employing *S*_*map*_ as cost function.

Remarkably, the CG mappings singled out by this optimization workflow were discovered to more likely retain atoms directly related to the biological function of the proteins of interest, thus linking the described information-theoretical approach to the properties of biological systems. It follows that this protocol represents not only a practical way to select the most informative mapping in a macromolecular structure, but also a promising paradigm to employ CGing as a controllable filtering procedure that can highlight relevant regions in a system.

The downside of the approach developed in Giulini et al. (2020) is its non-negligible computational cost, which is due to two factors:

1. The protocol requires in input a set of configurations of the high-resolution system that are sampled through an MD simulation, a computationally expensive task.
2. The stochastic exploration of the set of possible CG mappings is limited and time consuming due to the algorithmic complexity associated to *S*_*map*_ calculations.

The ultimate aim of this work is, thus, the development and assessment of a protein-specific machine learning model able to swiftly predict the mapping entropy arising from a reduction in the number of degrees of freedom employed to describe the system.

### Protein Structures and Data Sets

The DGN-based mapping entropy prediction model developed in this study is applied to two proteins extracted from the set investigated in Giulini et al. (2020), namely *(i)* *6d93*, a 31 residues long mutant of *tamapin*—a toxin of the Indian red scorpion (Pedarzani et al., 2002)—whose outstanding selectivity toward the calcium-activated potassium channels SK2 made it an extremely interesting system in the field of pharmacology (Mayorga-Flores et al., 2020); and *(ii)* *4ake*, the open conformation of *adenylate kinase* (Müller et al., 1996). This 214-residues enzyme is responsible for the interconversion between adenosine triphosphate (ATP) and adenosine diphosphate + adenosine monophosphate (ADP + AMP) inside the cell.

**FIGURE 3:**
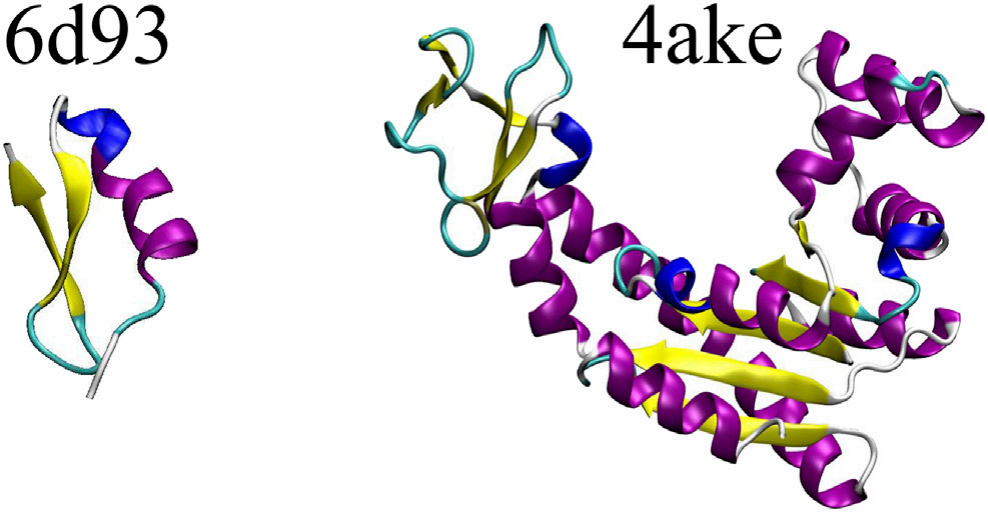
Protein structures employed in this work: the tamapin mutant (PDB code: 6d93) and the open conformation of adenylate kinase (PDB code: 4ake). The former, although small, possesses all the elements of proteins’ secondary structures, while the latter is bigger in size and has a much wider structural variability.

Figure 3 shows a schematic representation of *6d93* and *4ake*. Both proteins were simulated in explicit solvent for 200 ns in the canonical ensemble by relying on the GROMACS 2018 package (Spoel et al., 2005). For a more detailed discussion of these two molecules and the corresponding MD simulations, please refer to Sec. II.B and II.D of Giulini et al. (2020).

We train the machine learning model of each protein on a data set containing the molecular structure—the first snapshot of the MD trajectory—and many CG representations, the latter being selected with the constraint of having a number of retained sites equal to the number of amino acids composing the molecule. The data sets combine together randomly selected CG mappings (respectively, 4,200 for *6d93* and 1,200 for *4ake*) and optimized ones (768 for both systems). The corresponding mapping entropy values are calculated through the protocol described in Giulini et al. (2020).

Optimized mappings are obtained from independent Simulated Annealing (SA) Monte Carlo runs (Kirkpatrick et al., 1983; Černỳ, 1985): starting from a random selection of retained atoms, *S*_*map*_ is minimized for a defined number of steps after which the current mapping is saved and included in the data set. More specifically, at each step of a SA run we randomly swap a retained and a non-retained atom in the CG representation, compute *S*_*map*_, and accept/reject the move based on a Metropolis criterion. The SA effective temperature *T* decays according to $T\left(i\right)=T_{0}e^{-i/v}$, where *i* is the SA step and the parameters *v* and *T*_0_ are equal to those employed in Giulini et al. (2020). The 768 SA runs of each protein are divided into four groups of 192 elements depending on their length, respectively, 2 × 10^4^ (full optimization, as in Giulini et al. (2020)), 1 × 10^4^, 5 × 10^3^, and 2.5 × 10^3^ steps.

**FIGURE 4:**
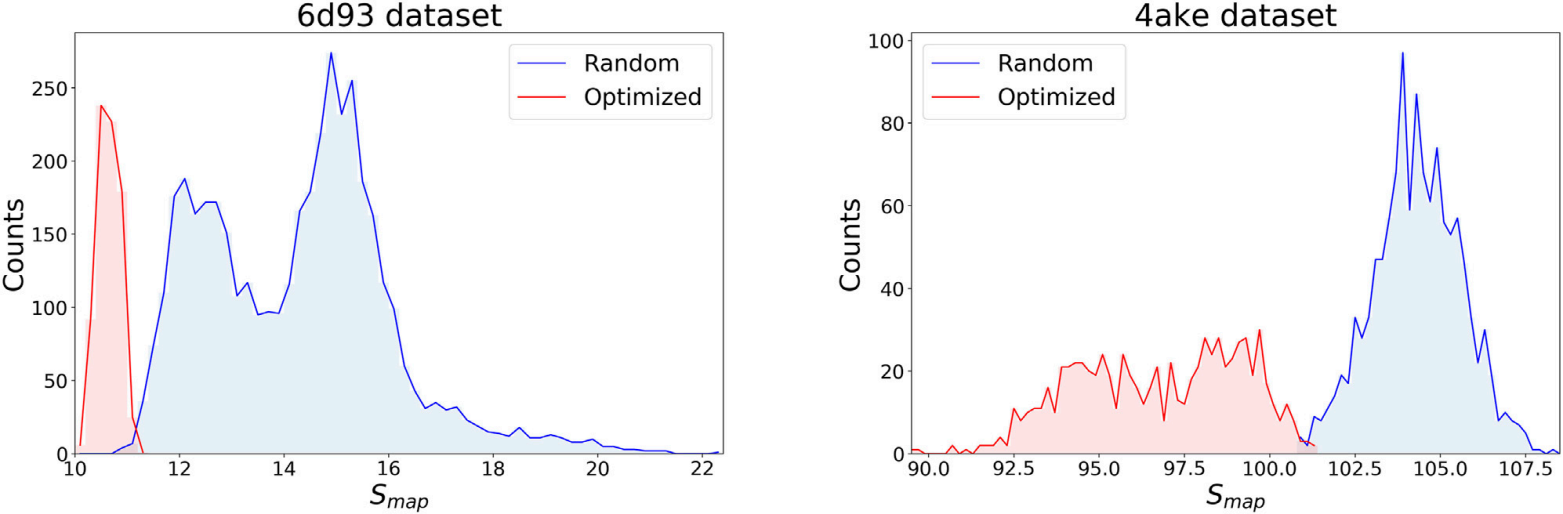
Distributions of target values for both data sets, *6d93* **(left)** and *4ake* **(right)**. For each protein, *S*_*map*_ data are displayed in two distinct, non-overlapping histograms depending on their origin: blue curves are filled with random instances, while red histograms represent optimized CG mappings. All values of *S*_*map*_ are in kJ/mol/K.

Figure 4 displays the distribution of *S*_*map*_ values in the data sets separately for the two systems, discriminating between random (blue) and optimized (red) CG mappings. In both structures the two curves have a negligible overlap, meaning that the set of values spanned by the optimized CG representations cannot be reached by a random exploration of the mapping space, i.e., this region possesses a very low statistical weight. A comparison of the *S*_*map*_ distribution of the two proteins, on the other hand, highlights that the mapping entropy increases with the system’s size: while the range of values covered has similar width in the two cases, the lower bound in mapping entropy of *4ake* differs of roughly one order magnitude from that of *6d93*.

For each analyzed protein, in Table 1 we report the computational time required to perform the MD simulation and a single *S*_*map*_ estimate. We note that the time associated with the calculation of *S*_*map*_ for a single CG mapping through the algorithm discussed in Giulini et al. (2020) grows from 2 to 8 minutes while moving from *6d93* to *4ake*. It is worth stressing that the proteins studied here are small, so that this value would dramatically increase in the case of bigger biomolecules.

**TABLE 1:** Computational cost of all-atom MD simulations and mapping entropy calculations for the two investigated proteins. Specifically, *MD CPU time* (respectively, *MD walltime*) represents the core time (respectively, user time) necessary to simulate the system for 200 ns on the GROMACS 2018 package (Spoel et al., 2005). Both *6d93* and *4ake* runs were performed on Intel Xeon-Gold 5118 processors, respectively, using 16 and 48 cores. *Single measure* is the amount of time that is required to compute, on a single core of the same architecture, the *S*_*map*_ of a given CG mapping by relying on the algorithm introduced in Giulini et al. (2020).

Protein	MD CPU time	MD walltime	Single measure
Tamapin (PDB code 6d93)	40.7 days	2.55 days	≃2.1 mins
Adenylate kinase (PDB code 4ake)	153.9 days	3.20 days	≃8.0 mins

### Data Representation and Machine Learning Model

**FIGURE 5:**
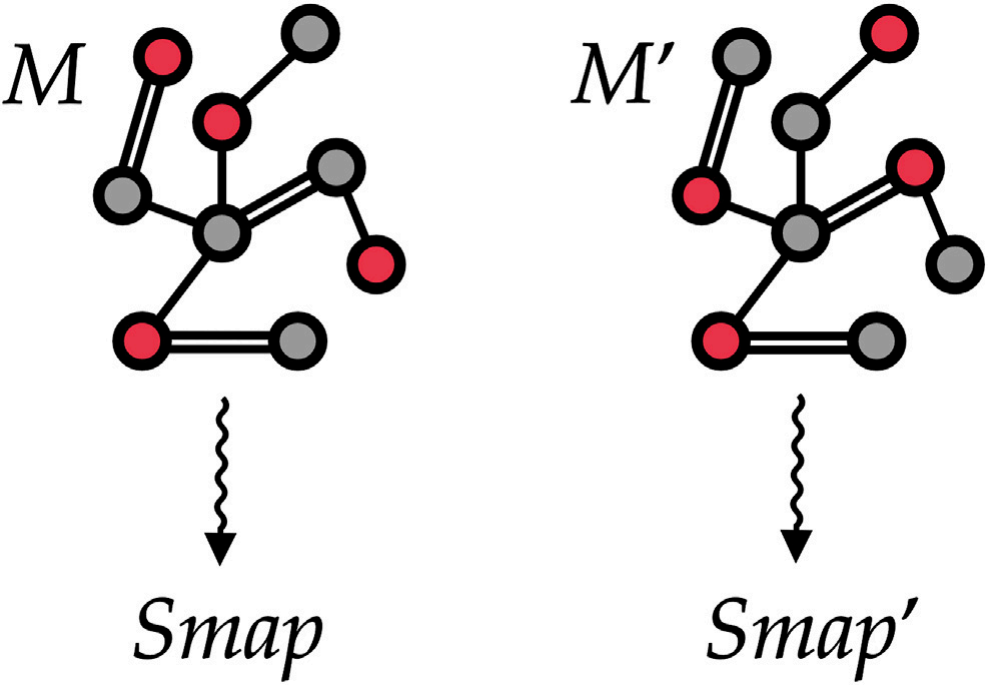
Two different mappings *M* and *M*ʹ associated with the same (schematic) protein structure. To train our machine learning model, we treat each protein as a graph where vertices are atoms and edges are placed among atoms closer than a given threshold. The selected CG sites in each of the two mappings are marked in red and encoded as a vertex feature. Our goal is to automatically learn to associate both mappings to proper values *S*_*map*_ and $S_{map}^{\text{′}}$ of the mapping entropy.

We represent each investigated protein structure as a static graph, see Figure 5. A graph *g* can be formally defined as a tuple $\left(v_{g},{ℰ}_{g}\right)$, where $v_{g}$ is the set of vertices (i.e., the entities of interest) and ${ℰ}_{g}=\left\{\left\{u,v\right\}|u,\;v\in v_{g}\right\}$ is the set of undirected edges (i.e., how entities are related). We define the neighborhood of a vertex *v* as the set of vertices connected to *v* by an edge, that is, $N_{v}=\left\{u\in v_{g}|\left\{u,\;v\right\}\in {ℰ}_{g}\right\}$. For the purpose of this work, each heavy atom composing the molecule corresponds to a vertex, and edges connect pairs of atoms that in the reference structure are closer than a selected threshold—in our case, 1 nm. At odds with other definitions of a CG site, the information about the decimation mapping can be directly encoded in the vertices of the protein graph by using a binary feature, with different selections of CG sites—an example being provided in Figure 5—corresponding to different values of *S*_*map*_. In addition, we enrich each vertex with 10 features, summarized in Table 2, which describe the physicochemical properties of the underlying atom; similarly, we consider the inverse atomic distance *e*_*uv*_ between vertices *u* and *v* as an edge feature.

**TABLE 2:** Binary features (0/1) used to describe the physicochemical properties of an atom in the protein, i.e., a vertex in the graph representation of the latter. In this simple model, we only provide the DGN with the chemical nature of the atom and of its residue, together with the flag *Bkb* that specifies if the atom is part of the backbone of the polypeptide chain.

Feature name	Description
C	Carbon atom
N	Nitrogen atom
O	Oxygen atom
S	Sulfur atom
HPhob	Part of a hydrophobic residue
Amph	Part of a amphipathic residue
Pol	Part of a polar residue
Ch	Part of a charged residue
Bkb	Part of the protein backbone
Site	Atom selected as a CG site

Once the protein structure and the CG mapping data sets are converted into this graph-like format (statistics in Table 3), we employ DGNs (Bacciu et al., 2020) with the aim of learning the desired property, namely the mapping entropy *S*_*map*_.

**TABLE 3:** Basic statistics of the data sets fed to the machine learning model. For each protein, we report the number of vertices (i.e., heavy atoms) in its graph representation, the total number of edges connecting them, and the average number of edges per vertex (Avg. degree). We also report the total number of CG representations of known mapping entropy provided in input to the protocol (Samples), including random and optimized ones.

Protein	Vertices	Edges	Avg. degree	Samples
*6d93*	230	21,474	93	4,968
*4ake*	1,656	207,618	125	1,968

**FIGURE 6:**
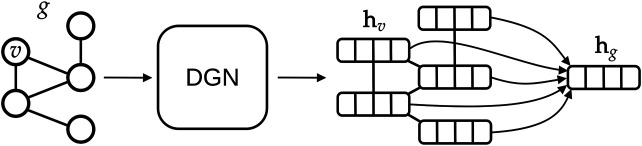
High-level overview of typical deep learning methodologies for graphs. A graph *g* is given as input to a Deep Graph Network, which outputs one vector, also called embedding or state, for each vertex *v* of the graph. In this study, we aggregate all vertex states *via* a (differentiable) permutation-invariant operator, i.e., the mean, to obtain a single state that encodes the whole graph structure. Then, the graph embedding is fed into a machine learning regression model (in our case a linear model) to output the *S*_*map*_ value associated with *g*.

The main advantages of DGNs are their efficiency and the ability to learn from graphs of different size and shape. This is possible for two reasons: first, DGNs focus on a local processing of vertex neighbors, so that calculations can be easily distributed; secondly, in a way that is similar to Convolutional Neural Networks for images (LeCun et al., 1995), DGNs stack multiple layers of graph convolutions to let vertices efficiently exchange information. The output of a DGN is a vector for each vertex of the graph, as sketched in Figure 6, and these can be aggregated to make predictions about a graph class or property. Again, we remark that the efficiency of the DGN is especially important in our context, where we want to approximate the complex *S*_*map*_ computational process in a fraction of the time originally required.

The main building block of a DGN is the “graph convolution” mechanism. At each layer *ℓ*, the DGN calculates the new state of each vertex *v*, i.e., a vector $h_{v}^{\ell +1}\in {\mathbb{R}}^{K}$, as a function of *v*’s neighboring states $h_{N_{v}}^{\ell }=\left\{h_{u}^{\ell }\in {\mathbb{R}}^{K}|u\in N_{v}\right\}$, where K∈ℕ is an hyperparameter of the model.

In general, a graph convolutional layer first applies a permutation-invariant function to the neighbors of each vertex, such as the sum or mean. The resulting aggregated vector is then passed to a multi-layer perceptron (MLP) that performs a nonlinear transformation of the input, thus producing the new vertex state $h_{v}^{\ell +1}$.

**FIGURE 7:**
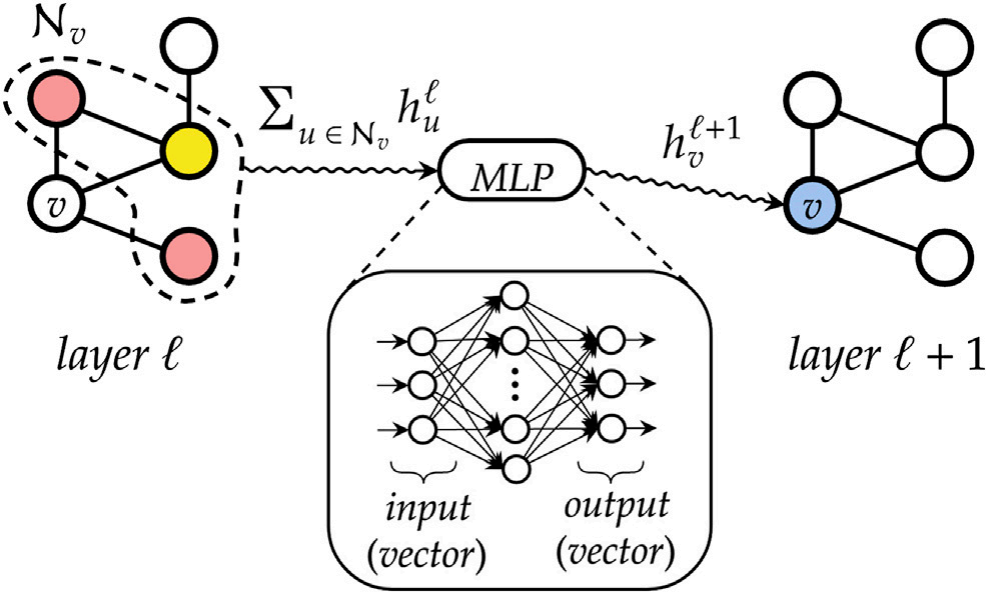
A simplified representation of how a graph convolutional layer works. First, neighboring states of each vertex *v* are aggregated by means of a permutation-invariant function, to abstract from the ordering of the nodes and to deal with variable-sized graphs. Then, the resulting vector is fed into a multi-layer perceptron that outputs the new state for node *v*.

In this study, we employ an extended version of the GIN model (Xu et al., 2019) or, equivalently, a restricted version of the Gated-GIN model (Errica et al., 2020) to consider edge attributes while keeping the computational burden low. Our graph convolutional layer can be formalized as follows:

$$h_{v}^{\ell +1}=MLP^{\ell }\left[\left(1+\epsilon^{\ell }\right)\text{×}h_{v}^{\ell }+\sum_{u\in N_{v}}h_{u}^{\ell }\text{×}e_{uv}\right],$$ (5)

where × denotes element-wise scalar multiplication, $\epsilon^{\ell }\in \mathbb{R}$ is an adaptive weight of the model, and *e*_*uv*_ is the scalar edge feature holding the inverse atomic distance between two atoms *u* and *v*. A pictorial representation of the transition between layer *ℓ* and layer *ℓ* + 1 is presented in Figure 7.

A few remarks about Eq. 5 are in order. First, the initial layer is implemented with a simple nonlinear transformation of the vertex features, that is, $h_{v}^{1}=MLP^{1}\left(x_{v}\right)$, where **x**_*v*_ is the vector of 10 features associated with each node (see Table 2); secondly, at each layer *ℓ*, we apply the *same* nonlinear transformation $MLP^{\ell }$ to all the nodes (i.e., a graph traversal), which allows us to treat variable-size graphs. Finally, the MLP weights are not shared across different layers, meaning that we train a different MLP for each layer. It is worth noting that this weight-sharing scheme at each layer resembles the one employed in Convolutional Neural Networks, where the same adaptive filter is applied to all the pixels in an image.

When building a Deep Graph Network, we usually stack *L* graph convolutional layers, with L∈ℕ being another hyperparameter, until the model produces a final state for each vertex. We call this state **h**_*v*_; in addition, we compute a global graph state **h**_*g*_ by aggregating all vertex states (see Figure 6). Being in vectorial form, **h**_*g*_ can then be fed to standard machine learning models to solve graph regression or classification tasks.

To produce a prediction ${\hat{S}}_{map}$, we first need to process and aggregate all node states into a single graph representation. In this work, we take into account the importance of selected (respectively, unselected V) CG sites $v_{g}^{s}\subset v_{g}$ (respectively, $v_{g}^{n}$) with a scalar adaptive weight *w*_*s*_ (respectively, *w*_*n*_). The resulting formula is

$${\hat{S}}_{map}=w_{out}^{T}\left\{\sum_{u\in V_{g}^{s}}\left[\left(h_{u}^{1},\ldots ,h_{u}^{L}\right)\text{×}w_{s}\right]+\sum_{u\in V_{g}^{n}}\left[\left(h_{u}^{1},\ldots ,h_{u}^{L}\right)\text{×}w_{n}\right]\right\},$$ (6)

where $w_{out}\in {\mathbb{R}}^{K*L}$ is a set of parameters to be learned, while square brackets denote concatenation of the different vertex states computed at different layers.

In particular, we use *L* = 5 layers and implement each $MLP^{\ell }$ as a one-layer feed-forward network with *K* = 64 hidden units followed by an element-wise rectifier linear unit (ReLU) activation function (Glorot et al., 2011). As the number of weights, without considering the bias, of $MLP^{\ell }$ is *K* ^2^ $\left(10\text{*}K\;\mathrm{for}\;MLP^{1}\right)$, the total number of weights in our architecture is $10\text{∗}K+K^{2}\text{∗}\left(L-1\right)+\left(L*K\right)+\left(L-1\right)+2=\mathrm{17350}$.

The loss objective used to train the DGN is the mean absolute error. The optimization algorithm is Adam (Kingma and Ba, 2015) with a learning rate of 0.001 and no regularization. We trained for a maximum of 10,000 epochs with early stopping patience of 1,000 epochs and mini-batch size 8, accelerating the training using a Tesla V100 GPU with 16 GB of memory.

To assess the performance of the model on a single protein, we first split the corresponding data set into training, validation, and test realizations following an 80%/10%/10% hold-out strategy. We trained and assessed the model on each data set separately. We applied early stopping (Prechelt, 1998) to select the training epoch with the best validation score, and the chosen model was evaluated on the unseen test set. The evaluation metric for our regression problem is the coefficient of determination (or R^2^ score).

### Wang–Landau Sampling

Figure 4 highlights how an attempt of detecting the most informative CG representations of a protein—i.e., those minimizing *S*_*map*_—through a completely unbiased exploration of its mapping space would prove extremely inefficient, if not practically pointless. Indeed, such optimized CG representations live relatively far away in the left tails of the *S*_*map*_ distributions obtained from random sampling, thus constituting a region of exponentially vanishing size within the broad mapping space. It would then be desirable to design a sampling strategy in which no specific value of *S*_*map*_ is preferred, but rather a *uniform coverage* of the spectra of possible mapping entropies—or at least of a subset of it, vide infra—is achieved.

To obtain this “flattening” of the *S*_*map*_ landscape we rely on the algorithm proposed by Wang and Landau (WL) (Wang and Landau, 2001a; Wang and Landau, 2001b; Shell et al., 2002; Barash et al., 2017). In WL sampling, a Markov chain Monte Carlo (MC) simulation is constructed in which a transition between two states *M* and *M*ʹ —in our case, two mappings containing *N* sites but differing in the retainment of one atom—is accepted with probability

$$W\left(M\to M^{\prime }\right)=\text{min}\left\{1,\frac{{\text{Ω}}_{N}\left[S_{map}\left(M\right)\right]}{{\text{Ω}}_{N}\left[S_{map}\left(M^{\prime }\right)\right]}\right\}.$$ (7)

In Eq. 7, ${\text{Ω}}_{N}\left(S_{map}\right)$ is the number of CG representations with *N* retained sites exhibiting a mapping entropy equal to *S*_*map*_, that is, the mapping entropy’s density of states,

$${\text{Ω}}_{N}\left(S_{map}\right)=\sum_{M}\delta \left[N\left(M\right),N\right]\delta \left[S_{map}\left(M\right),S_{map}\right],$$ (8)

where the sum is performed over all possible CG representations of the system.

When compounded with a symmetric proposal probability *T* for the attempted move, $T\left(M\to M^{\prime }\right)=T\left(M^{\prime }\to M\right)$, the Markov chain defined in Eq. 7 generates, at convergence, CG representations distributed according to $P\left(M\right)\propto \;1/{\text{Ω}}_{N}\left[S_{map}\left(M\right)\right]$ (Wang and Landau, 2001a; Wang and Landau, 2001b). As the equilibrium probability of visiting a mapping is proportional to the inverse of the *S*_*map*_ density of states, the WL simulation results in a flat histogram of sampled mapping entropies *over the whole range of possible ones*.

Critically, the density of states ${\text{Ω}}_{N}\left(S_{map}\right)$ is a priori unknown and is itself a byproduct of the WL scheme. ${\text{Ω}}_{N}\left(S_{map}\right)$ is self-consistently constructed by means of a sequence *k* = 0,...*K* of nonequilibrium simulations that provide increasingly accurate approximations to the exact result, iterations being stopped when a predefined precision is achieved.

Having divided the range of possible values of the mapping entropy in bins of width *δS*_*map*_, the WL self-consistent protocol is based on three quantities: the overall density of states ${\text{Ω}}_{N}\left(S_{map}\right)$, the histogram of sampled mapping entropies at iteration *k*, *H*_*k*_ (*S*_*map*_), and the modification factor $f_{k}$ governing convergence–for *k* = 0, one typically initializes ${\text{Ω}}_{N}\left(S_{map}\right)=1$ for each value of *S*_*map*_ and $f_{0}=e$.

At the beginning of WL iteration *k*, the histogram *H*_*k*_ (*S*_*map*_) is reset. Subsequently, a sequence of MC moves among CG mappings driven by the acceptance probability presented in Eq. 7, is performed. If a transition between two CG representations *M* and *Mʹ*— respectively with mapping entropies *S*_*map*_ and *S*_*map*′_ predicted by the trained DGNs—is accepted, the entries of the histogram and density of states are updated according to

$$H_{k}\left(S_{map}^{\text{′}}\right)=H_{k}\left(S_{map}^{\text{′}}\right)+1,$$ (9)

$${\text{Ω}}_{N}\left(S_{map}^{\text{′}}\right)=f_{k}\times {\text{Ω}}_{N}\left(S_{map}^{\text{′}}\right).$$ (10)

In case the move $M\to M^{\prime }$ is rejected, one has to replace $S_{map}^{\text{′}}$ with *S*_*map*_ in Eqs. 9, 10.

The sequence of MC moves is stopped—that is, iteration *k* ends—when *H*_*k*_ (*S*_*map*_) is “flat”, meaning that each of its entries does not exceed a threshold distance from the average histogram $\langle H_{k}\rangle$: a typical requirement is $p_{flat}\times \langle H_{k}\rangle <H_{k}\left(S_{map}\right)<\left(2-p_{flat}\right)\times \langle H_{k}\rangle$ for every value of *S*_*map*_, *p*_*flat*_ being the selected flatness parameter. At this stage, WL iteration k+1 begins with a reduced modification factor, where we set $f_{k+1}=\sqrt{f_{k}}$.

Convergence of the self-consistent scheme is achieved when $f_{k}\approx 1$ —more precisely, when $\text{ln}\left(f_{k}\right)$ becomes smaller than a predefined value $\text{ln}\left(f_{end}\right)$. Up to a global multiplicative factor, the resulting density of states ${\text{Ω}}_{N}\left(S_{map}\right)$ reproduces the exact result with an accuracy of order $\text{ln}\left(f_{end}\right)$ (Landau et al., 2004).

In order to avoid numeric overflow of ${\text{Ω}}_{N}\left(S_{map}\right)$ along the WL simulation, we consider its logarithm ${\text{Σ}}_{N}\left(S_{map}\right)={\text{lnΩ}}_{N}\left(S_{map}\right)$. Starting from Eq. 7, the acceptance probability $W\left(M\to M^{\prime }\right)$ expressed in terms of Σ reads

$$W\left(M\to M^{\prime }\right)=\text{min}\left\{1,\text{exp}\left[{\text{Σ}}_{N}\left(M\right)-{\text{Σ}}_{N}\left(M^{\prime }\right)\right]\right\},$$ (11)

while within iteration *k* of the self-consistent scheme, the update prescription of Σ after an (accepted) MC move—see Eq. 10—becomes

$${\text{Σ}}_{N}\left(S_{map}^{\text{′}}\right)={\text{Σ}}_{N}\left(S_{map}^{\text{′}}\right)+\text{ln}\left(f_{k}\right).$$ (12)

Finally, in a logarithmic setup, the modification factor $\text{ln}\left(f_{k}\right)$ follows the simple reduction rule $\text{ln}\left(f_{k+1}\right)=\text{ln}\left(f_{k}\right)/2$, with $\text{ln}\left(f_{0}\right)=1$.

The WL algorithm in principle enables the reconstruction of the density of states of an observable over the whole range of possible values of the latter; at the same time, knowledge of the sampling boundaries proves extremely beneficial to the accuracy and rate of convergence of the self-consistent scheme (Wüst and Landau, 2008; Seaton et al., 2009). In our case, for each analyzed protein, such boundaries would correspond to the minimum and maximum achievable mapping entropies $S_{map}^{min}$ and $S_{map}^{max}$ in the space of all CG representations of the system obtained by retaining *N* of its constituent atoms. As this information is *a priori* unknown, in our implementation of the WL algorithm we limit the range of explorable values of *S*_*map*_ by rejecting all MC moves $M\to M^{\prime }$ for which $S_{map}^{\text{′}}<S_{map}^{min}$ or $S_{map}^{\text{′}}>S_{map}^{max}$, in each system setting $S_{map}^{min}$ and $S_{map}^{max}$ as, respectively, the minimum and maximum values of the mapping entropy in the corresponding data set. Note that for each protein $S_{map}^{min}$ is the outcome of a thorough optimization procedure, and can thus be considered a reasonable approximation of the system’s *absolute* minimum of the mapping entropy. Imposing an upper bound on *S*_*map*_ through $S_{map}^{max}$, on the other hand, simply amounts at requiring the WL sampling algorithm not to visit uninteresting regions of the mapping space of each biomolecule, that is, CG representations characterized by a huge amount of information loss with respect to the all-atom reference. The values of $S_{map}^{min}$ and $S_{map}^{max}$ employed for the two proteins investigated in this study are presented in Table 4, together with the input parameters required by the WL protocol—the bin size *δS*_*map*_, the convergence modification factor $\text{ln}\left(f_{end}\right)$, and the flatness parameter *p*_*flat*_.

**TABLE 4:** Set of parameters employed for the WL exploration of the mapping entropy space for both analyzed proteins. $\text{ln}\left(f_{0}\right)$ and $\text{ln}\left(f_{end}\right)$ respectively represent the modification factor at the beginning and at the end of the self-consistent scheme in a logarithmic setup, see Sec.*Wang–Landau Sampling*. *p*_*flat*_ is the minimal histogram flatness required to halve the modification factor; with *p*_*flat*_ = 0.8, all bins in the histogram *H* (*S*_*map*_) must have a population between 0.8 and 1.2 times its average 〈H〉. *range* is the interval of permitted values of the mapping entropy in the WL scheme, while *δS*_*map*_ is the bin size employed for its discretization. Both *range* and *δS*_*map*_ are expressed in kJ/mol/K.

Parameter	*6d93*	*4ake*
ln(f_0_)	1	1
ln(f_end_)	10^−6^	10^−6^
p_flat_	0.8	0.8
*range*	[10−22.4]	[89.4−108.6]
δS_map_	0.2	0.2

## Results and Discussion

We first analyze the results achieved by DGNs in predicting the mapping entropy associated to a choice of the CG representation of the two investigated proteins; specifically, we employ the R^2^ score as the main evaluation metric and the mean average error (MAE) as an additional measure to assess the quality of our model in fitting *S*_*map*_ data. The R^2^ scores range from −∞ (worst predictor) to 1 (best predictor).

Table 5 reports the R^2^ score and MAE in training, validation, and test. We observe that the machine learning model can fit the training set and has excellent performances on the test set. More quantitatively, we achieve extremely low values of MAE for *6d93*, with an R^2^ score higher than 0.95 in all cases. The model performs slightly worse in the case of *4ake*: the result of R^2^ = 0.84 on the test set is still acceptable, although the gap with the training set (R^2^ = 0.92) is non-negligible.

**TABLE 5:** Results of the machine learning model in predicting the mapping entropy on the training (TR), validation (VL), and test (TE) sets for the two analyzed proteins. We display both the R^2^ score and the mean average error (MAE, kJ/mol/K).

Protein	TR MAE	TR R^2^	VL MAE	VL R^2^	TE MAE	TE R^2^
*6d93*	0.13	0.99	0.33	0.95	0.33	0.96
*4ake*	0.91	0.92	1.2	0.85	1.35	0.84

**FIGURE 8:**
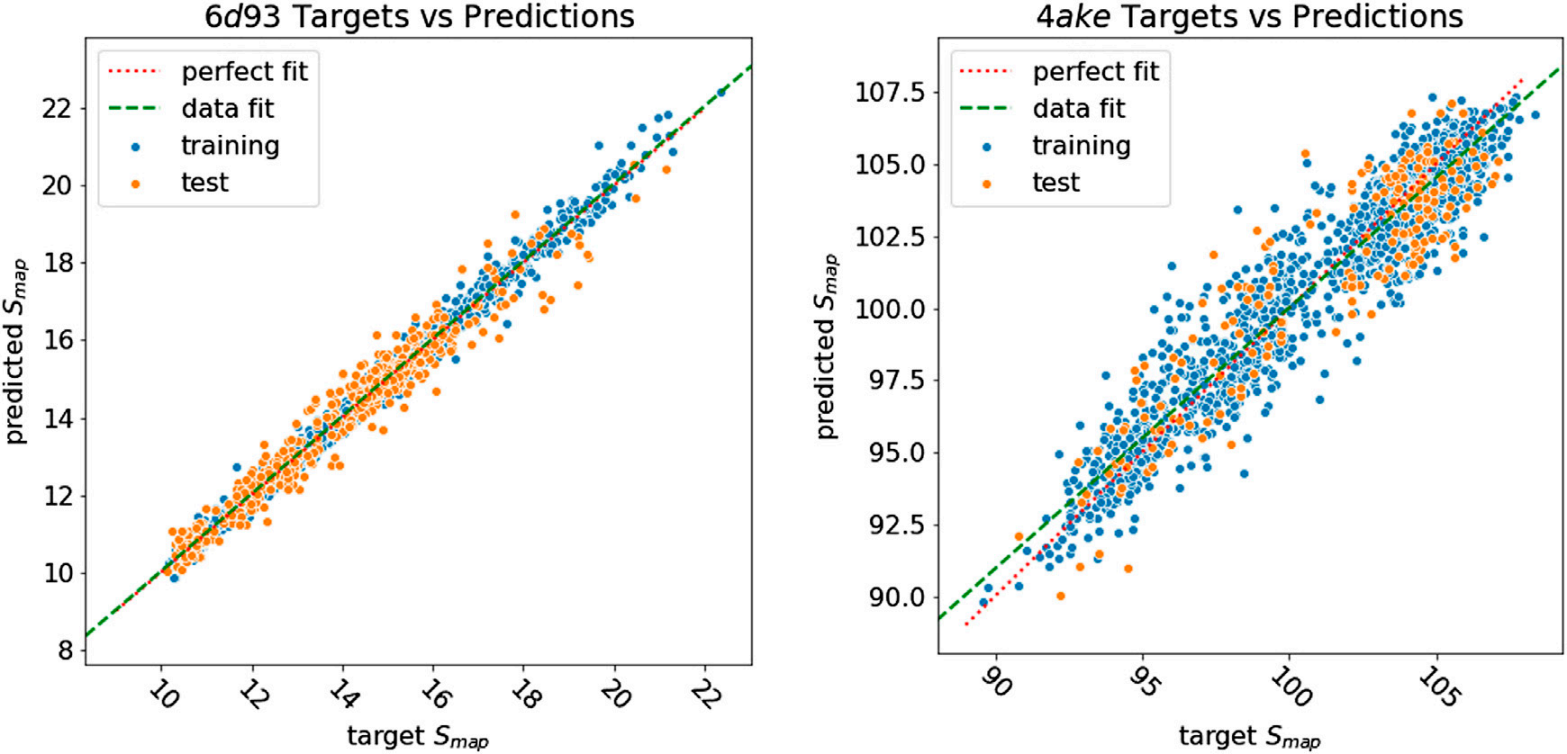
Plot of *S*_*map*_ target values against predictions of all samples for *6d93* **(left)** and *4ake* **(right)**. Training samples are in blue, while test samples are in orange. A perfect prediction is represented by points lying on the red dotted diagonal line (perfect fit). To show that in the case of 4*ake*, the model slightly overestimates the *S*_*map*_ of optimized mappings and underestimates the rest, we include in the plot the green dashed line obtained by fitting a linear model on the data (data fit). All values of *S*_*map*_ are in kJ/mol/K.

Figure 8 shows how predicted values for training and test samples differ from the ground truth. Ideally, a perfect result corresponds to the point being on the diagonal dotted line. We can see how close to the true target are both training and test predictions for *6d93*. The deviation from the ideal case becomes wider for *4ake*, but no significant outlier is present. A more detailed inspection of the *4ake* scatter plot in Figure 8, on the other hand, reveals that the network tends to slightly overestimate the value of *S*_*map*_ of optimized CG mappings for $S_{map}≲100\;\;kJ/\text{mol}$, whereas the opposite is true for $S_{map}≳100\;\;kJ/\text{mol}$, where random CG mapping values are mildly underestimated.

The dissimilarity in performance between the two data sets is not surprising if one takes a closer look at their nature. In fact, as highlighted in Figure 3, adenylate kinase is both larger and more complex than the tamapin mutant, and the CG mapping data set sizes are very different due to the heavy computational requirements associated with the collection of annotated samples for *4ake*. As a consequence, training a model for *4ake* with excellent generalization performance becomes a harder task. What is remarkable, though, is the ability of a completely adaptive machine learning methodology to well approximate, in both structures, the long and computationally intensive algorithm for estimating *S*_*map*_ of Giulini et al. (2020). Critically, this is achieved only by relying on a combination of static structural information and few vertex attributes, that is, in absence of a direct knowledge for the DGNs of the complex dynamical behavior of the two systems as obtained by onerous MD simulations.

The computational time required by the machine learning model to perform a single *S*_*map*_ calculation is compared to the one of the algorithm presented in Giulini et al. (2020) in Table 6. As the protocol of Giulini et al. (2020) relied on a CPU machine, we report results for both CPU and GPU times. Overall, we observe that inference of the model can speed up mapping entropy calculations by a factor of two to five orders of magnitude depending on the hardware used. Noteworthy, these improvements do not come at the cost of a significantly worse performance of the machine learning model. In addition, this methodology is easily applicable to other kinds of molecular structures, as long as a sufficiently large training set is provided as input.

**TABLE 6:** Comparison between the time required to compute the *S*_*map*_ of a single CG mapping through the algorithm presented in Giulini et al. (2020) and the inference time of the model (CPU as well as GPU). For both proteins, CPU calculations were performed on a single core of a Intel Xeon-Gold 5118 processor, while GPU ones were run on a Tesla P100 with 16 GB of memory. The machine learning model generates a drastic speedup, enabling a wider exploration of the *S*_*map*_ landscape of each system.

Protein	Single measure	Inference GPU (CPU)	Time ratio GPU (CPU)
*6d93*	≃2.1 mins	≃0.9(98.7) ms	≃140000×(1276×)
4*ake*	≃8.0 mins	≃4.8(1103.2) ms	≃100000×(435×)

By embedding the trained networks in a Wang–Landau sampling scheme, see Wang–Landau sampling, we are able to retrieve the density of states ${\text{Ω}}_{N}\left(S_{map}\right)$ defined in Eq. 8 for *6d93* and *4ake*, that is, we can estimate the number of CG representations throughout the mapping space of each protein that exhibits a specific amount of information loss with respect to the all-atom reference. We stress that reaching convergence of the self-consistent WL protocol required to probe approximately 4.8 × 10^6^ and 3 × 10^7^ CG representations for *6d93* and *4ake*, respectively: such an extensive sampling is only made feasible by the computational gain provided by the machine learning model.

**FIGURE 9:**
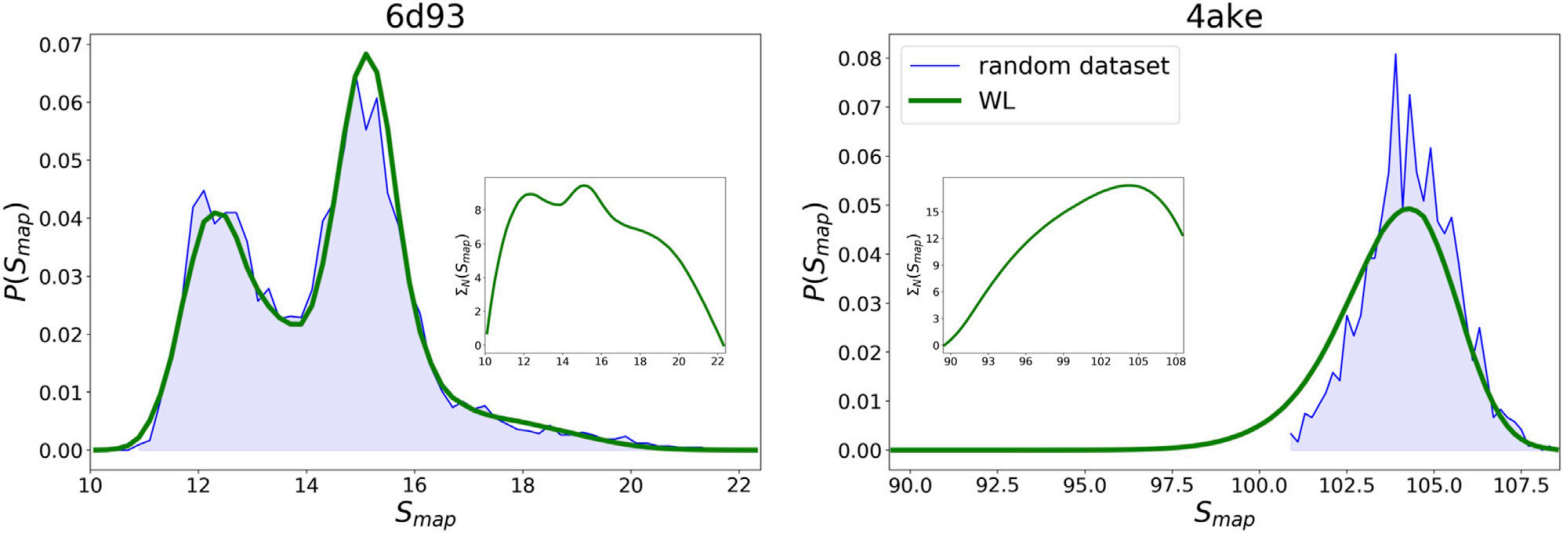
Comparison between the probability densities $P\left(S_{map}\right)$ for the two systems estimated *via* the Wang–Landau algorithm enhanced by the DGNs (green lines) and the distributions generated by a random sampling of mappings (blue areas). In inset, the logarithm of the WL density of states, $\text{Σ}\left(S_{map}\right)$, is reported, after a scaling that assigns to the Σ of the most scarcely populated bin the value of zero. All values of *S*_*map*_ are in kJ/mol/K.

WL predictions for the logarithm of the density of states ${\text{Σ}}_{N}\left(S_{map}\right)={\text{lnΩ}}_{N}\left(S_{map}\right)$ of the two proteins are presented in Figure 9. As for *6d93*, we observe the presence of a steep increase of Σ starting from low values of the mapping entropy, followed by two main peaks respectively located at $S_{map}\approx 12.5$ and 15 kJ/mol/K. After the second peak Σ decreases, exhibiting a shoulder for high mapping entropies. On the other hand, the Σ of *4ake* displays a relatively gradual growth toward its unique maximum, the latter being located at $S_{map}\approx 105\;kJ/\text{mol}/K$, before starting to decrease.

Given the WL ${\text{Ω}}_{N}\left(S_{map}\right)$—or equivalently ${\text{Σ}}_{N}\left(S_{map}\right)$—it is possible to calculate the probability $P\left(S_{map}\right)$ of observing a particular mapping entropy by performing a completely random exploration of the space of CG representations of a system,

$$P\left(S_{map}\right)=\frac{{\text{Ω}}_{N}\left(S_{map}\right)}{\sum_{S_{map}}{\text{Ω}}_{N}\left(S_{map}\right)}.$$ (13)

Results for the $P\left(S_{map}\right)$ of *6d93* and *4ake* are shown in Figure 9. In the case of *6d93*, we note that the WL sampling scheme produces a probability density that is fully compatible with the (normalized) histograms of Figure 4. In particular, the WL graph resembles the histograms in Figure 4 if we remove the nonrandom, optimized instances whose statistical weight is negligible. This result is highly nontrivial, as it proves that the trained DGN of *6d93* does not overfit the training set and is able to predict the correct population of the true mapping entropy landscape.

As regards *4ake*, the agreement between the two curves presented in Figure 9 is still remarkable, though not as precise as in the case of *6d93*. More quantitatively, the left tail of the probability density predicted by the WL scheme is shifted of roughly 1 kJ/mol/K toward lower values of *S*_*map*_ with respect to the distribution obtained from random sampling. This mismatch can be ascribed to the mild overfitting problem observed in Figure 8: the network has the tendency to underestimate (respectively, overestimate) the value of *S*_*map*_ associated with random (respectively, optimized) CG representations, resulting in an increase in the predicted population of mappings at the intersection of the two sets.

## Conclusion and Perspectives

Molecular dynamics simulations constitute the core of the majority of research studies in the field of computational biophysics. From protein folding to free energy calculations, an all-atom trajectory of a biomolecule gives access to a vast amount of data, from which relevant information about the system’s properties, behavior, and biological function is extracted through an *a posteriori* analysis. This information can be almost immediate to observe (even by naked eye) and quantify in terms of few simple parameters–e.g., the process of ligand binding can be seen in a graphical rendering of the trajectory and made quantitative in terms of the distance between ligand and protein; much more frequently, though, it is a lengthy and nontrivial task, tackled through the introduction of complex “filtering” strategies, the outcomes of which often require additional human intervention to be translated in intuitive terms (Tribello and Gasparotto, 2019; Noé et al., 2020).

A protocol aiming at the unsupervised detection of the relevant features of a biomolecular system was recently proposed (Giulini et al., 2020). The method relies on the concept of mapping entropy *S*_*map*_ (Shell, 2008; Rudzinski and Noid, 2011; Shell, 2012; Foley et al., 2015), that is, the information that is lost when the system is observed in terms of a subset of its original degrees of freedom: in Giulini et al. (2020), a minimization of this loss over the space of possible reduced representations, or CG mappings, enabled to single out the most informative ones. By performing a statistical analysis of the properties of such optimized mappings, it was shown that these are more likely to concentrate a finer level of detail—so that more atoms survive the CG’ing procedure—in regions of the system that are directly related to the biological function of the latter. The mapping entropy protocol thus represents a promising filtering tool in an attempt of distilling the relevant information of an overwhelmingly complicated macromolecular structure; furthermore, this information can be immediately visualized and interpreted as it consists of specific subsets of atoms that get singled out from the pool of the constituent ones. Unfortunately, estimating the *S*_*map*_ associated with a specific low-resolution representation is a lengthy and computationally burdensome process, thus preventing a thorough exploration of the mapping space to be achieved along the optimization process.

In this work, we have tackled the problem of speeding up the *S*_*map*_ calculation procedure by means of deep machine learning models for graphs. In particular, we have shown that Deep Graph Networks are capable of inferring the value of the mapping entropy when provided with a schematic, graph-based representation of the protein and a tentative mapping. The method’s accuracy is tested on two proteins of very different size, a tamapin mutant (31 residues) and adenylate kinase (214 residues), with a R^2^ test score of 0.96 and 0.84, respectively. These rather promising results have been obtained in a computing time that is up to five orders of magnitude shorter than the algorithm proposed in Giulini et al. (2020).

The presented strategy holds the key for an extensive exploration of the space of possible CG mappings of a biomolecule. In fact, the combination of trained networks and Wang–Landau sampling allows one to characterize the mapping entropy landscape of a system with impressive accuracy.

The natural following step would be to apply the knowledge acquired by the model on different protein structures, so that the network can predict values of *S*_*map*_ even in the absence of an MD simulation. As of now, however, it is difficult to assess if the information extracted from the training over a given protein trajectory can be fruitfully employed to determine the mapping entropy of another, by just feeding the structure of the latter as input. More likely one would have to resort to database-wide investigations, training the network over a large variety of different molecular structures before attempting predictions over new data points. In other words, obtaining a transfer effect among different structures by the learning model may not be straightforward, and additional information could be needed to achieve it. Analyses on this topic are on the way and will be the subject of future works.

In conclusion, we point out that the proposed approach is completely general, in that the specific nature and properties of the mapping entropy played no special role in the construction of the deep learning scheme; furthermore, the DGN formalism enables one to input graphs of variable size and shape, relaxing the limitations present in other kinds of deep learning architectures (Giulini and Potestio, 2019). This method can thus be transferred to other problems where different selections of a subset of the molecule’s atoms give rise to different values of a given observable (see e.g., Diggins et al., 2018) and pave the way for a drastic speedup in computer-aided computational studies in the fields of molecular biology, soft matter, and material science.

## Data Availability

The data sets employed for this study and the code that performs the Wang Landau-based exploration of the mapping space are freely available at https://github.com/CIML-VARIAMOLS/GRAWL.
